# At the cross-roads of participatory research and biomarker discovery in autism: the need for empirical data

**DOI:** 10.1186/s12910-015-0082-0

**Published:** 2015-12-15

**Authors:** Afiqah Yusuf, Mayada Elsabbagh

**Affiliations:** Department of Psychiatry, McGill University, Ludmer Research & Training Building, 1033 Pine Avenue West, Montreal, QC H3A 1A1 Canada

**Keywords:** Autism, Community-based participatory research, Biological markers

## Abstract

**Background:**

Identifying biomarkers for autism can improve outcomes for those affected by autism. Engaging the diverse stakeholders in the research process using community-based participatory research (CBPR) can accelerate biomarker discovery into clinical applications. However, there are limited examples of stakeholder involvement in autism research, possibly due to conceptual and practical concerns. We evaluate the applicability of CBPR principles to biomarker discovery in autism and critically review empirical studies adopting these principles.

**Methods:**

Using a scoping review methodology, we identified and evaluated seven studies using CBPR principles in biomarker discovery.

**Results and conclusions:**

The limited number of studies in biomarker discovery adopting CBPR principles coupled with their methodological limitations suggests that such applications are feasible but challenging. These studies illustrate three CBPR themes: community assessment, setting global priorities, and collaboration in research design. We propose that further research using participatory principles would be useful in accelerating the pace of discovery and the development of clinically meaningful biomarkers. For this goal to be successful we advocate for increased attention to previously identified conceptual and methodological challenges to participatory approaches in health research, including improving scientific rigor and developing long-term partnerships among stakeholders.

## Background

A biomarker is a stable and objective indicator of biological state. The search for biomarkers associated with autism spectrum disorder (ASD) is currently underway [1–3]. Among the potential uses of biomarkers are the following: biomarkers for susceptibility indicate a predisposition for a condition such as the presence of a genetic variant associated with autism [4]. *Presymptomatic* biomarkers can identify an individual developing the condition before overt behavioural symptoms are noticeable. For example, brain imaging techniques such as electroencaphalography are being researched to help distinguish infants at higher risk for autism before behavioural symptoms manifest [5]. Biomarkers can also be used as a *diagnostic* tool: in autism, chromosomal microarray analysis has been recommended as a first-tier diagnostic test for autism to supplement behavioural diagnostic procedures, clarifying the presence of diagnosable genetic conditions [6, 7]. Finally, biomarkers may also be *prognostic* i.e. to predict the outcome of a condition to allow for tailored and targeted treatments. All of these biomarkers have the potential to accelerate detection of ASD and access to tailored services for improved outcomes for those with ASD and their families.

There are challenges in translating research on biomarkers in the clinic [8]. Firstly, what precisely should these biomarkers map onto in ASD and at which developmental time-point? Considering the condition’s heterogeneity [9–12] and its developmental nature [13] deciding on the impairment at a certain time-point to which a biomarker can predict is challenging. Secondly, a person’s position on the spectrum is not fixed throughout life [14–16], making biomarker measurement sensitive to a person’s development. Finally, discovered biomarkers thus far have poor sensitivity and specificity when applied to the general population, limiting their utility in community-based care [8].

Such challenges in translating biomarker discovery to clinical applications have been often deliberated in bioethics. This includes discussion of ethical issues related to bio-banking, prenatal or population screening, and direct-to-consumer testing [17–19]. We have previously argued that issues related to translating biomarker discovery are much broader at the intersection of scientific, social, and health systems challenges [8]. Effective knowledge translation in this area relies on engagement of key stakeholder groups, the intended beneficiaries of this research.

Engaging stakeholders in this area would inform researchers of questions relevant to stakeholders. Thus, end products of this research would address stakeholders’ needs and circumvent potential harms, accelerating appropriate translation of biomarker discoveries. However, the extent to which stakeholders are engaged in biomarker discovery is currently unclear and some have suggested that biomarker discovery is on the whole discordant with stakeholders’ needs [20, 21]. Our goal is to systematically evaluate these claims.

To achieve this goal, we adopt a framework of community-based participatory research (CBPR) as it relates to autism biomarker discovery. CBPR aims to support active engagement of the community in research through equitable and sustainable partnerships between researchers and a unit of community [22]. Given that theoretical debates presented have been extensive, we focus on two areas that have not been sufficiently elaborated on before. First, we evaluate the potential challenges in CBPR application to biomarker discovery. Secondly, we systematically assess the quantity and quality of *empirical* biomarker discovery studies that adopt CBPR principles. We begin with a brief overview of CBPR.

### Principles of community-based participatory research

CBPR encompasses a range of approaches that support community involvement in research. These approaches can operate in a wide range of research designs and involve diverse groups and populations [23]. In CBPR, both community and researcher enter into an equitable partnership to address research questions deemed relevant to the target community [22, 24].

Historically, the roots of CBPR can be traced back to two periods: 1) research aimed to bridge the theory-practice gap and 2) efforts to empower marginalized groups when the academic institution was critiqued as being too distanced from social problems [25]. The WK Kellogg Community Health Scholars Program was the first to conceptualize a common definition of CBPR, leading to CBPR “foundational books” [24–26]. Within this program, Israel et al. [27] outlined nine specific core principles of CBPR: recognizing a “community” as a unit of identity, leveraging on strengths and resources of both the community and researchers, facilitating collaborative, equitable, and long-term involvement of all partners in a cyclical and iterative manner in all phases of research [27]. CBPR also promotes co-learning and empowering that attends to social inequalities and addressing health from both positive and ecologic perspectives while balancing investments into knowledge and intervention for mutual benefit of all partners. These principles are considered ideal goals to strive toward, concretely built on the following core components of CBPR [28]:

1. Forming a CBPR partnership
2. Assessing community strengths and dynamics
3. Identifying priority local health concerns and research questions
4. Designing and conducting etiologic, intervention, and/or policy research
5. Feeding back and interpreting the findings
6. Disseminating and translating research findings
7. Maintaining, sustaining, and evaluating the partnership

The applications of CBPR are far-reaching and flexibly adapted based on the type of research [23]. The nature of “partnerships” of the community can vary from community representatives acting as an advisory board to researchers [24], to the community directing research projects with researchers only providing technical expertise [29]. CBPR also differs on the intended outcome of the partnership: some partnerships grew to overcome a community’s distrust of research groups from previous negative experiences [30], while others were developed to enhance the uptake of findings in the community [23, 31].

### Conceptual challenges to CBPR as they apply to biomarker discovery in autism

Despite apparent advantages, CBPR poses significant and previously recognized conceptual challenges in health research. These challenges include ensuring proper representation of a community [32], forecasting long-term impacts of basic research [33, 34], and threats to internal validity, specifically concerns for decreased randomization and contamination [35]. We consider how each challenge relates to biomarker discovery and its respective solution.

#### Stakeholder definition

Among the most frequently recognized challenges to CBPR is how to adequately represent the “community” [35–37]. In participatory research, there is a potential danger of selection bias, where highly outspoken subgroups may not represent the broader population [35]. We have previously discussed the complexity of defining ‘stakeholders’ in autism research, noting that ‘stakeholders’ have diverse needs, may benefit from research advances differently, and have varying interest in involvement in research [38].

In the case of biomarker discovery, strong claims of an inherent conflict between researchers and ‘stakeholders’ are presented often without defining who the ‘stakeholders’ are [20, 39]. Homogenizing the stakeholder group is unhelpful because needs faced by individuals with autism and their families are as heterogeneous as the condition itself [8] and moderated by contextual, geographic, and socio-cultural factors [40, 41].

Views differ on what autism even is: some advocate groups endorsed a search for a “cure” for autism, a view *neurodiversity* proponents find objectionable because autism is considered part of “natural human variation” [8, 42]. When perceived by some stakeholders as a means to find a “cure”, biomarker discovery becomes much more controversial [8]. On the other hand, biomarker discovery is considered a research priority to a geographically and culturally diverse group of stakeholders if such biomarkers were to facilitate identification and timely access to care [40, 41]. Moreover, it seems likely that biomarker discovery is a more relevant priority depending on the developmental pathway of the person affected: parents of children suspected of or recently diagnosed with autism may be more likely to find value in prospective, diagnostic, or prognostic biomarkers relative to families whose children have been diagnosed for several years.

Therefore, stakeholder representation is a major challenge in public engagement in autism research in general [38], applicable to biomarker discovery. The term *stakeholder* is context-dependent, does not always mean *beneficiary* from research and not *all* stakeholders will want to be or can be adequately represented and engaged [38]. For successful stakeholder engagement to be achieved, a systematic assessment of priorities, needs, and experiences of the stakeholder group is needed.

#### Forecasting consequences

The second major challenge for CBPR relevant for biomarker discovery lies in foreseeing potential social consequences of any discovery. This is due to the difficulty in predicting the potential ripple effects of understanding the mechanism of a gene, and the fact that knowledge advances in incremental steps by building upon previous discoveries. This general challenge in health research [43] has been previously recognized in autism biomarker discover. On the one hand, premature involvement of the community in a new discovery before it has proven clinical value would inflate public expectations, leading to a subsequent loss of public trust in science when these hyped promises are not met [8]. On the other hand, the *lack* of community engagement has been shown to lead to a loss of public trust in science.

An example comes from the national partnership with Aboriginal representatives, where community representatives expressed that “Aboriginal communities have been researched to death” and unequivocally objected to any further research [44]. After extensive deliberation, representatives agreed that a health survey would be acceptable, but only with an equitable partnership between Aboriginal representatives and researchers in the project. Similarly, Arbour and Cook presented examples of respectful genetic research under the concept of “DNA on loan” in understanding the mechanism of a rare chromosomal abnormality in a First Nations community [30]. The group first discussed the research priorities with the family to develop trust with them. Participating families were then kept updated with the research progress. Later the group facilitated informed health care and counseling for each family based on their findings. The community and family themselves determined whether or not the specification of First Nations could be used to promote health in the wider community, thus allowing the community to weigh the possibility of stigma with the potential benefit of research for others.

These examples illustrate that the challenge of forecasting research results can be mitigated through active consideration of research priorities and process with participating families. Such involvement also led to benefits for both groups: the community protected themselves from possible stigma and received informed care and counseling, while the research group was able to address their research question and advance knowledge in their field.

#### Internal validity

A third major challenge of participatory research is the threat to internal validity. A systematic review of CBPR showed that despite the wealth of studies adopting this approach, limited studies have reported a complete intervention with authors only detailing either their findings or study methodology [35]. This made it difficult to conclude if participatory research is associated with low scientific quality [35]. Nevertheless, concerns of decreased randomization and contamination in CBPR remain [35, 45].

This problem is faced especially in randomized controlled trials (RCTs), a methodology with clearly defined standards of scientific rigor. In RCTs, participants randomly allocated to the control group must not be exposed to the intervention and vice versa, causing *contamination*. Because members of the community work closely with each other and with researchers on the research project in CBPR, the likelihood for decreased randomization and contamination among individuals is high [45].

However, the disappointing results of some non-participatory “high-rigor” RCTs have shown that the improved adherence to interventions found in CBPR along with the unintended benefits of partnerships may outweigh the concern for these threats, all of which can also be mitigated. Authors of a well-designed nationwide RCT (see COMMIT trial [46]) admitted that the lack of community involvement in their trial was the biggest contributing factor in its disappointing outcome, and that “an exclusive focus on risk factors alone may be inappropriate” [47]. In contrast, Andrews et al. worked closely with the community in a tobacco cessation intervention and implemented randomization at the community level to reduce the risk of reduced randomization and contamination [48]. Because of the partnership, they not only showed promising outcomes on smoking cessation, they also reported high retention rates (87 %) and improved self-efficacy for both the community health workers implementing the intervention and the participants receiving the intervention [49]. Therefore, as proposed for other areas of health research [35], CBPR has the potential to enhance the quality of conventional research methods for biomarker discovery in autism by increasing participation rate, lowering drop-off over time where applicable, and increasing capacity for the community and individual to adopt the findings, such as a new test or an intervention.

Taken together, the above considerations suggest that participatory research is neither conceptually nor methodologically in conflict with biomarker discovery in autism. Nevertheless, the extent to which there is underlying conflict between biomarker discovery specifically and participatory research is a question that continues to be debated [21].

Despite the wealth of theoretical positions and arguments, what remains largely unknown is whether principles of CBPR have already been adopted in biomarker discovery. Understanding how to achieve participation in this field can resolve these theoretical debates and contribute to future development of biomarker discovery [38].

## Methods

We use scoping review methodology to address the question of how to achieve participation in biomarker discovery. A scoping review methodology [50, 51] allows the mapping of key concepts of participation to a complex research area. Unlike systematic reviews that focus on a narrow question with specific study designs decided a priori, our use of scoping review methodology would include a broader range of study designs and help us understand the state of the evidence in a field. Therefore, the selected methodology is appropriate considering the lack of knowledge on what exists in participatory approaches in autism biomarker research.

The goal of the study is to identify previous applications of CBPR in autism biomarker discovery. Because the state of the science of CBPR in biomarker discovery is unknown and may be limited, we adopt a broad but systematic definition of CBPR as articulated by Israel et al. [27]. While not all principles are suitable to all forms of stakeholder engagement, there is general agreement that these principles are likely to characterize ideal partnerships.

Studies were included if they met each of the following criteria:

1. The study should involve one or more of the following key stakeholder groups: a person with ASD, families of individuals with ASD, professionals in the field of ASD, or policy makers. While we had these categories of stakeholders a priori, we did not specifically limit the search strategy targeting specific groups to have a higher chance of retrieving relevant articles;
2. To increase the chances of retrieving possible studies, “involvement” of stakeholder groups in research was defined broadly. This would also capture any study that performed *community assessment and diagnosis*. Thus, any study that obtained individuals’ views regarding biomarker discovery that could potentially be used to shape future research would be included;
3. The topic of discussion in the study included biomarkers for identification and intervention in autism, including but not limited to genetic testing and brain imaging;
4. The study was conducted in English;
5. Original empirical research (quantitative or qualitative) were included.

Exclusion criteria were:

1. Reviews and opinion papers;
2. The study involved childhood conditions other than ASD;
3. The study only assessed associations between potential biomarkers and ASD symptoms;
4. The study reported on collaboration between any of the above key stakeholder groups and researchers on topics other than biomarkers for ASD e.g. in implementing interventions.

We searched Medline, CINAHL, PsycINFO, and Embase using a comprehensive list of search terms. Author lists and references were also cross-referenced for potentially relevant articles. Once key articles were identified, we retrieved a list of articles that cited those key articles as well. The search was completed on June 17^th^, 2014.

Articles were screened first by title and abstract based on the above inclusion and exclusion criteria. Screened articles were retrieved for full text articles. Using a data extraction form, we extracted relevant study characteristics (e.g. document classification, study design, method employed, study population, main outcomes). For quantitative studies, we employed a *narrative method*, wherein results of studies were compiled and organized to form a “composite” understanding of the current state of knowledge [52]. For qualitative studies, the data extraction form contained codes that were recursively applied [53]. In other words, we first developed an initial coding scheme based on the nine CBPR principles articulated by Israel et al. [27]. The initial coding scheme consisted of broad categories namely definition of community, outcomes for collaboration, the stage of research at which collaboration occurred, and the evaluation of collaboration. We revised the coding scheme appropriately after applying it to an article; for example, articles that elaborated on the definition of community required additional codes such as community attitudes, awareness and needs. These additional codes were then retroactively applied to all articles [53]. This work conformed to the Declaration of Helsinki and was approved by McGill University Institutional Review Board.

## Results

**Fig. 1:**
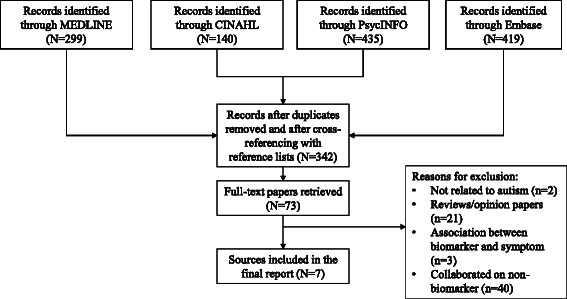
Flow diagram of the progression of inclusion of articles

Our search yielded a total of 342 studies (Fig. 1). After screening the articles first by title and abstract, 73 articles were retrieved for full-text assessment. Out of these 73, only seven examples of original research studies fit with our inclusion criteria. While all possible types of biomarker testing were targeted in the search strategy, all studies captured except for one focused on genetic testing. Similarly, while all possible stakeholders were considered, studies have only involved parents of children with autism.

A synthesis of these seven studies yielded three themes that correspond closely to previously articulated principles and components of CBPR [22, 27]. The first is “assessing community strengths and dynamics” by identifying stakeholder attitudes and expectations towards the application of autism biomarkers in their lives. The second is “identifying priority local health concerns and research questions” by collaborating with stakeholders in setting long-term priorities for autism research. The third is in “designing and conducting etiologic research” by collaboration among stakeholders in devising an imaging protocol. In what follows, we synthesize and critically review identified evidence for each of these themes.

### Identification of stakeholder perceptions and attitudes

While community assessment by itself is not CBPR, it is an essential component in establishing partnerships with the community prior to initiating CBPR, and in line with one principle of CBPR in defining the community of interest by “assessing community strengths and dynamics” [27]. Considering the dearth of CBPR in the field of biomarker discovery in general, and in the field of autism biomarkers in particular, we considered what empirical work has previously focused on *community assessments*.

We identified four studies that examined community attitudes towards one biomarker measurement for ASD: genetic testing. They mainly surveyed and/or interviewed parents of children with autism on their attitudes towards genetic testing, and their experience in and motivation for undergoing testing. On the whole, many parents are ‘supportive’ of genetic testing for ASD. Using semi-structured interviews (*n* = 42), Chen et al. found that 69 % of parents favoured genetic testing for ASD [54]. Another study using an Internet survey (*n* = 25) showed that 80 % of parents with a child with ASD would want their younger undiagnosed child tested for an increased risk for ASD even if the test could not confirm or rule out a diagnosis [55]. Those who supported genetic testing do so because they believe it would support early intervention and treatment, help families understand the etiology of autism, and inform family planning [54]. Parents specifically noted that genetic testing could alleviate guilt and promote acceptance of the condition [56].

While many were in favour of genetic testing, those against did not foresee its value because they perceived ASD as “incurable”, they perceived no impact for genetic testing in the child’s lives or for future pregnancies, and they have no family history of ASD [57]. Another study found the fear of stigma from testing was also another reason for opposing it [55].

Taken together, these studies have obtained perspectives on genetic testing for autism through qualitative and quantitative methodologies. Several limitations of the studies hinder the generalizability of their findings. Despite multiplicity of stakeholders in autism, only parents were the focus. Two of the studies relied on genetics research databases for recruitment, thus there is the possibility of a biased sample. The studies represented views of stakeholders on genetic testing within potentially very different contexts: research versus clinical practice. Moreover, the use of Internet surveys is useful in capturing a large number of respondents, but the survey instrument may only reflect items of interest to the researchers. While two of the studies employed semi-structured interviews, their reporting of results lacks methodological coherence, reflexivity of researcher’s role, and method of triangulation [58, 59].

In sum, while some studies have attempted to empirically examine stakeholder perspectives, these studies are characterized by methodological limitations, which impacted the generalizability of the findings. To draw valid conclusions about the perspectives of stakeholder groups on biomarker discovery, improved attention to recruitment methods and clarity of constructs assessed are vital.

### Collaborative priority-setting

No research projects in autism biomarker discovery have yet to involve participants in determining research questions directly. However, in line with the CBPR principle of recognizing priorities identified by the community [27], two studies focused on obtaining long-term priorities for autism research from the community [21, 60]. In addition to student group discussions, Higashijima et al. invited a diverse array of community members to monthly public science cafes through a variety of recruitment methods that included parents of children attending nurseries catering to ASD, and employees of childcare services. These community members discussed their views on the relationship between autism research and society with the guiding question, “Towards the construction of an autism-friendly society, what is the most impressive/important thing for you after finishing the conversation in today’s café discussion?” Discussion points were then analyzed to produce a list of topics that the community would want to discuss further. This list was then used to develop a survey, which was sent out to the larger community to further refine the discussion priorities. Survey results were then presented to researchers in a 3-day event dedicated to foster a discussion on creating an “autism-friendly society.” Points included in the final social agenda for autism research that needs further discussion included the following: ASD research for a cure, definition of ASD, issues surrounding ASD diagnosis and content for dissemination to the public.

Pellicano et al. employed focus groups and survey methodology to obtain views of 1,517 members of the autism community on the current landscape of autism research in the UK, along with their research priorities for the future for all fields of autism research [21]. Many participants called for a more balanced distribution of funding across the different research areas. When asked about their priorities for future autism research, participants endorsed research on support and services, efforts to improve public knowledge about autism, and of greater investment into autism research in general. The online survey showed that participants unanimously agreed that all research questions are important, with subtle differences (from “moderately important” to “very important”) between groups on specific questions. The research questions most frequently endorsed as important by autistic adults, practitioners, and family members are understanding how to improve life skills of autistic adults, how to meet their needs through public services, how autistic people think and learn, and on the future prospects for autistic adults.

The two studies outline the feasibility of setting priorities in a safe and open environment for both scientists and community members. Despite the relatively large samples, both studies present limitations. They employed convenience sampling to maximize participation, without formal consideration of representativeness to a target population, or even a specific definition of what target population was intended. Moreover, groupings of participants with the expectation that most within one group will share the same views is a potentially reductionist approach not reflective of real-world complexity. For example, a “researcher” group is not a meaningful category when properties of the researcher are unknown e.g. the researcher’s field of research, career stage, and focus populations.

Other potential risks to generalizability introduced by the two studies are ambiguous content and biased framing of the question. For example, one study asked participants if they were satisfied with the pattern of funding for research. Yet, the categories for research funding were presented as “biology” vs. “intervention”, potentially misleading community participants that research in one area is more likely than the other to directly lead to community benefits. Participants were asked to discuss their research priorities after being presented with the pattern of UK funding. It is possible that participants’ perspectives changed after deliberating over an investment “unbalance” in research funding.

Overall, the ongoing considerations highlight the complexity of surveying and/or deliberating research priorities. Notably absent is the use of well-established deliberation approaches abundantly used in global health research, such as the Delphi technique [61] and stakeholder dialogue [62].

### Collaboration in research design

One study we identified illustrates feasibility and value of collaboration with the community in design [63], in a way that strengthens conventional research methods and “building on strengths and resources within the community” [27]. Nordahl et al. collaborated with parents in designing a magnetic resonance imaging (MRI) protocol without the use of sedation for their child. Typically, imaging research in ASD requires children to be anesthetized to limit movement during imaging. As a result, imaging research without the use of sedation in ASD is limited to high-functioning children [64], who may not represent others on the spectrum. Nordahl et al. succeeded in obtaining quality scans of 93 % of their participants without sedation by collaborating with parents of children who participated. The research team first prepared a handout to describe the MRI protocol to the parents and child. With the parents, they then developed an individualized strategy guided by a questionnaire that assessed the child’s sleep patterns. They followed this individualized strategy during the experiment by recreating the child’s bedtime routine and sleeping environment at the imaging center. Parents reported positive feedback on the experience. This study demonstrates that it is feasible to include parents in the design of a study protocol and to ascertain feedback on their experience. Notably, such collaboration allows researchers to better address their research questions while catering to the immediate needs of families.

## Discussion

Our review has revealed limited albeit clear empirical examples of participatory research in autism biomarker discovery. Applications of CBPR have addressed three areas: 1) understanding of needs and priorities of a target community, 2) long-term priority setting with the community, and 3) collaboration in research design. Despite the fit of these empirical studies with CBPR principles, it appears surprising that their number is so limited given that biomarker discovery is an area of major investment and the emerging literature advocating the need for community engagement. What our review could not address is whether similar approaches have already been used in biomarker discovery but their findings not published.

What possible barriers might explain the lack of CBPR applications in biomarker discovery? One possible barrier is the sheer complexity of engagement in this area. The condition impacts a multitude of diverse communities, each with a complex group of stakeholders whose attitudes and perspectives are variable. What is clear based on our findings is that previously identified potential challenges of participatory research are more likely whenever the stakeholder group is not defined as a coherent and meaningful sample. Considering that conducting a *community assessment and diagnosis* prior to partnering with the community is a “key factor” in the success of carrying out community-based participatory research generally [22, 31, 65], defining the community of interest would then be an essential first step in adopting CBPR in autism biomarker discovery.

More generally, autism research is ‘reinventing the wheel’ in adopting participatory elements, instead of building on experiences and models in similar areas of health research. For example, a group of researchers working with small Alaskan native communities have proposed a CBPR-approach to conducting genetic studies for complex conditions (obesity, diabetes, and cardiovascular disease) and sharing these results to maximize the potential benefit and understanding [66]. The research team held open dialogue sessions between the researchers, the tribal council, and community representatives from their Yup’ik Eskimo study population, in which genetic education workshops were paired with discussions on Alaskan Native culture to create culturally respectful experimental protocols. Community involvement occurred throughout recruitment to prevent group harm and stigmatization. When results were ready, consultations with the community helped produce culturally relevant formats for presentations. Dissemination of the results was made in Yup’ik with the presence of at least one research member to answer questions. Such a format serves as an important framework from which biomarker discovery in autism could be feasibly adapted.

A further challenge is that the notion of ‘conflict’ between researchers and the community assumes a false distinction between two groups and suggests that one is expected to yield to the views of the other. Yet, advancement of knowledge does not occur in a vacuum – society is rarely impervious to its long-term benefits and detriments. Contributions by autistic researchers and advocates to debates on research priorities within mainstream scientific journals [67, 68] have also blurred the boundaries between “community” and “researcher”. Autism research is vulnerable to funder and public pressure that may undermine the value of scientific discovery and advancement of knowledge [8, 38]. Such pressures may be, in part, why scientific priorities were historically driven by what turned out to be simplistic promises, such as a “gene” for autism [8, 38].

Our findings suggest that available data are currently too limited to evaluate the claim that biomarker discovery is indeed misaligned with other stakeholder perspectives [20, 21]. Available studies were not only few, but also were characterized by methodological limitations impacting generalizability. When pushed towards polemics, conflicting priorities between *researchers* versus *the public* become a false notion lacking pragmatic real-world value.

## Conclusion

We suggest that deliberation around the intended outcomes of research, both short- and long-term, would instigate the same progress seen in other areas of health research, while mitigating concerns around participation. We propose that re-conceptualizing biomarker discovery in autism as participatory would entail clarifying and increasing its social relevance, enhancing rather than undermining its rigor, and accelerating its intended benefits to society. The success of this vision will rest on long-term partnerships among stakeholders to achieve enhanced public trust and engagement in science that would yield benefits to all involved.
